# A Comparative Study of the Presence of the Palmaris Longus Tendon Using Physical and Ultrasound Examination

**DOI:** 10.7759/cureus.85854

**Published:** 2025-06-12

**Authors:** Hasan Al-Ali, Nour Gamgoum, Michael Atalla, Andrew Yacoub, Ali Skiredj, Youssef Skiredj, Haider Hilal, Tarek Alambrouk, James Coey, Sadia Javed

**Affiliations:** 1 School of Medicine, St. George's University School of Medicine, True Blue, GRD; 2 Medical Imaging, University of Toronto, Toronto, CAN; 3 School of Medicine, St. George's University School of Medicine, Newcastle upon Tyne, GBR

**Keywords:** anatomical variability, msk ultrasound, palmaris longus muscles, palmaris longus tendon, tendon graft

## Abstract

Introduction

The palmaris longus muscle (PLM), located in the forearm's anterior compartment, plays an essential role in wrist flexion. Its tendon is often used for grafting because of its accessibility and minimal function. However, its anatomy varies, with congenital absence being the most common variation. This study aims to detect the incidence of the palmaris longus tendon (PLT) and compare physical examination methods with standard ultrasonography, as previous studies have only utilized physical examinations.

Methods

In this study, 61 participants were examined bilaterally (122 wrists) using three different physical examination methods: the Schaeffer, Thompson, and Mishra I tests. The ultrasonography test was conducted by a single observer using a GE LOGIQ e ultrasound system (GE HealthCare Technologies, Inc., Chicago, IL, USA), equipped with a linear transducer with a frequency range of 12-15 Hz, attached transversely to the anterior distal forearm. The tendon was visualized anterior to the median nerve, medial to the flexor carpi radialis, and superficial to the flexor retinaculum. To assess the difference between physical examination and ultrasound, statistical analyses were conducted on subgroups using a t-test. Additionally, PLT incidence was evaluated according to gender, ethnicity, and hand dominance.

Results

On ultrasound examination, the PLT was detected in 72.13% of wrists bilaterally (n = 88), 5.74% unilaterally (n = 7), and was absent in 22.13% of the 122 wrists examined (n = 27). Physical examination methods detected an average of 56.28% bilaterally (n = 68.66), 6.56% unilaterally (n = 8), and 37.16% as absent (n = 45.33). Total detection by ultrasound was 77.87% (n = 95), while the physical examination average was 62.84% (n = 76.66). Overall, there is a significant difference between the physical examination methods and ultrasonography in detecting PLT, with ultrasonography demonstrating greater accuracy. It should also be noted that the prevalence of PLT is not affected by gender.

Conclusion

Ultrasonography is crucial in clinical settings to confirm the presence of the PLT, even when a physical examination is inconclusive. Physical and ultrasound approaches can, therefore, be combined to avoid producing incorrect negative results when locating the PLT for tendon grafting.

## Introduction

The palmaris longus muscle (PLM), a slender, fusiform structure located in the superficial anterior compartment of the forearm, is a phylogenetically regressive muscle that plays a minimal role in wrist flexion. Despite its limited function, the palmaris longus tendon (PLT) holds significant clinical importance due to its frequent use as a donor graft in reconstructive and plastic surgeries, including tendon transfer procedures, ligament reconstructions, and ptosis corrections [1,2]. Its superficial location, anatomical simplicity, and negligible contribution to forearm functionality make it an ideal graft source [3].

However, the presence of the PLM is highly variable across populations. Congenital absence, either unilateral or bilateral, is the most commonly reported anatomical variation, with agenesis rates ranging up to 60%, depending on demographic and ethnic factors, as seen in a population in Turkey [4]. Identifying the presence or absence of the tendon would, therefore, be crucial in preoperative planning, particularly in procedures where tendon harvesting is considered.

Traditional identification of the PLT relies on various physical examination maneuvers, such as the Schaeffer, Thompson, and Mishra I tests, all of which involve specific wrist and finger positioning to highlight the tendon’s visibility or palpability [5]. While these tests are convenient and non-invasive, their diagnostic reliability is limited.

Ultrasonography, on the other hand, offers a non-invasive, real-time imaging modality capable of visualizing soft tissue structures with high resolution. Despite its advantages, few studies have compared ultrasonography directly with physical examination techniques in the context of PLT detection, where most studies refer to physical examination as a rapid way of identifying the presence without incorporating ultrasound, possibly due to its ease for large sample groups [1,2,4]. This highlights the need for further investigation into ultrasound's ability to identify the palmaris longus.

This study aims to assess the prevalence or absence of the PLT in a clinical sample and to compare the diagnostic accuracy of three physical examination methods with ultrasonography. By addressing the limitations of physical examination alone and emphasizing the utility of ultrasonographic confirmation, this research contributes to a more reliable and evidence-based approach for identifying the PLT in clinical and surgical practice.

## Materials and methods

Study design

In this diagnostic-oriented study, notices were used as a means to recruit participants across different stages of medical education at St. George’s University (SGU), Northumbria University (NU) campus in Newcastle upon Tyne, UK. Participating subjects included SGU/NU medical students in either the four-year medical program or the five- and six-year integrated pre-medical programs. In total, 61 students were recruited to participate, meaning 122 wrists were assessed separately for the presence or absence of the PLT. Before the data collection phase of the study, conducted in SGU/NU’s anatomy laboratory, subject consent was obtained through a verbal consent form, a participant consent form, and an information sheet. All three forms are available if necessary. This project was approved by the Ethical Review Board at NU (approval no. 2024-7087-7008).

In addition to the supplied forms, a participant questionnaire was given to each participant upon entry to the venue for the study. These questionnaires were utilized by both the participants and the researchers. On the sheet, the participants were asked to answer questions about their ethnicity, age, dominant hand, and whether they had undergone any surgical procedures on their hands and/or wrists in the past. All these data, in conjunction with the latter question used as an exclusion criterion, were essential to infer associations between such variables and the presence or absence of the PLT. The researchers of the study also used the questionnaire to indicate the presence or absence of PLT, following the physical examination maneuvers they performed. A copy of the participant questionnaire is attached in the Appendix section.

Protocol

With respect to the physical examinations, three separate stations were set up, each relating to a specific test: Schaeffer’s, Thompson’s, and Mishra I. Participants would move from station to station until they arrived at the fourth and final station: the confirmatory ultrasound. This final station aimed to validate the results of the physical examination maneuvers conducted in the three stations. Of utmost importance, each station was examined independently, with curtains that blocked sight and audition between stations to minimize observer bias. 

The first physical examination test, conducted in station 1, was the Schaeffer’s test, where participants opposed the thumb and fifth metacarpal, followed by wrist flexion. The second station was the Thompson’s test, where participants were asked to make a fist with the thumb positioned over most digits, followed by wrist flexion once again. In the third test station, the Mishra I test was used, where participants passively hyperextended their wrist, followed by active wrist flexion against resistance. In each of these three tests, a distinctive, superficial, and longitudinal mass would appear on the midline of the anterior wrist, medial to the flexor carpi radialis, that is both visible and palpable, giving a positive result. If no presence of the PLT was concluded, a tentative absence was noted.

**Figure 1:**
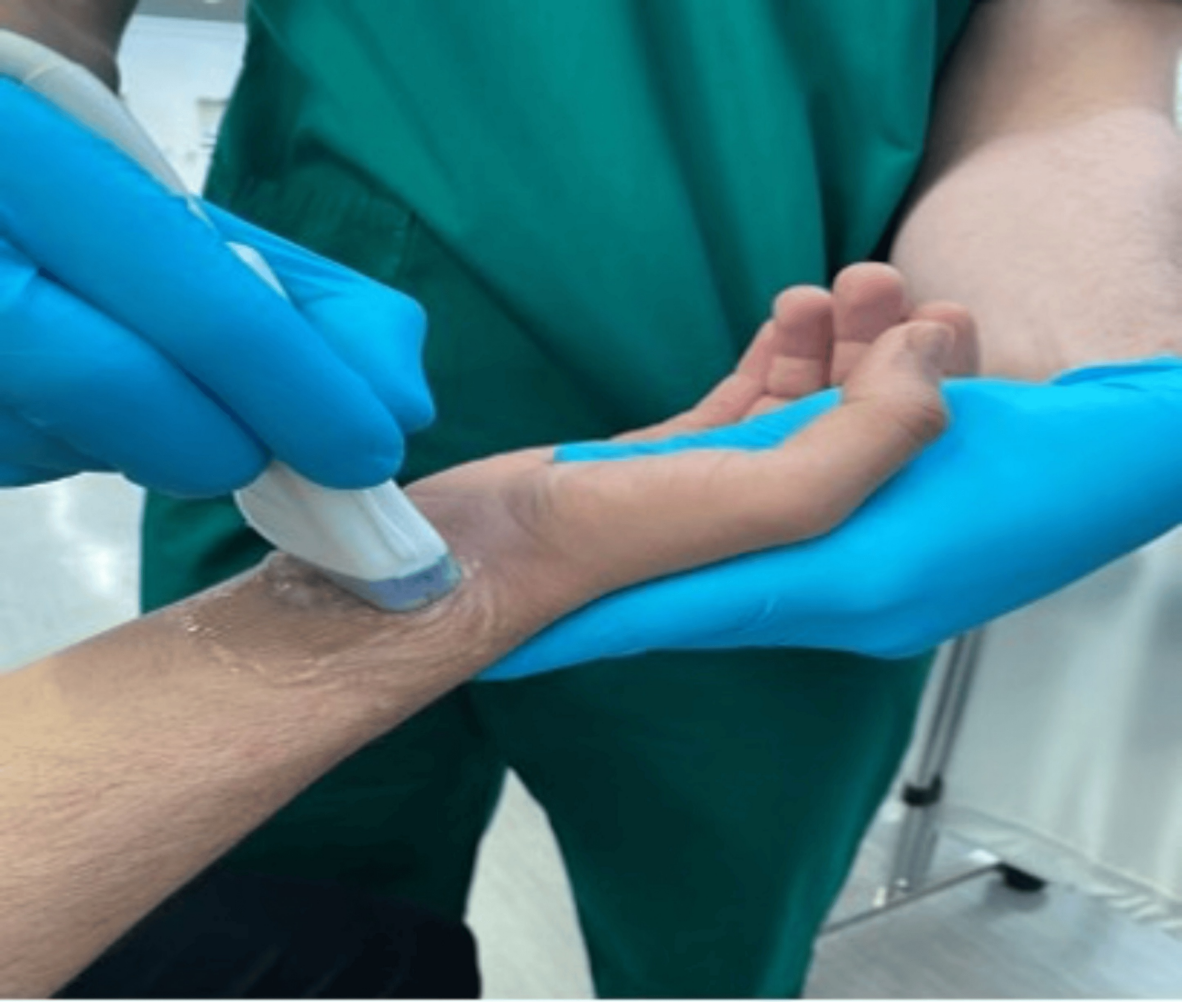
Ultrasound Probe Placement on the Wrist The linear transducer probe was aligned transversely on the anterior distal portion of the supported forearm, with a perpendicular orientation.

**Figure 2:**
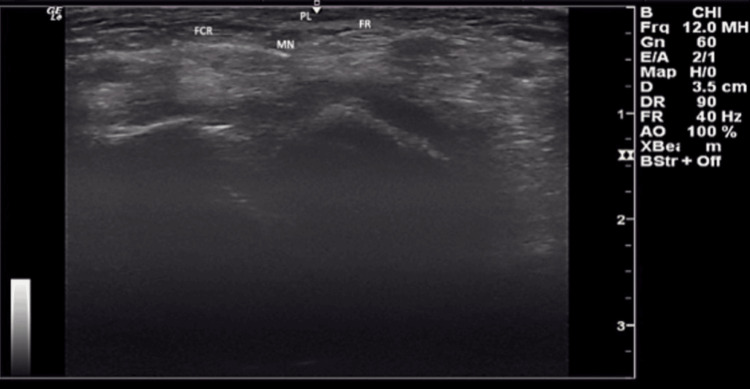
Confirmed Presence of Tendon by Ultrasound Palmaris longus (PL), as labeled in the figure, is identified in the anterior compartment of the forearm, superficial to the flexor retinaculum (FR). Relevant structures, including the flexor carpi radialis (FCR), visualized laterally, and the median nerve (MN), visualized posteriorly, are also shown.

**Figure 3:**
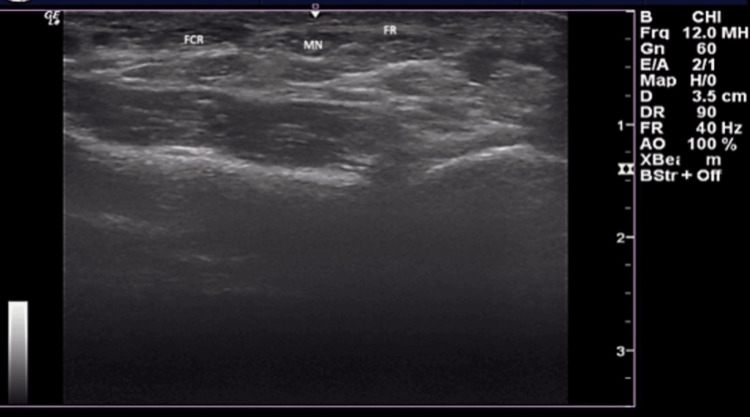
Confirmed Absence by Ultrasound Ultrasound did not detect the palmaris longus (PL) in this image. No presence of a fusiform muscle was noted superficial to the flexor retinaculum (FR). Additionally, relevant anatomical structures, such as the median nerve (MN) and flexor carpi radialis (FCR), are labeled in this figure.

The ultrasound station consisted of placing the probe on both wrists of the participant, subsequently, as depicted in Figure 1. This test was conducted by a single observer using a GE LOGIQ e ultrasound system (GE HealthCare Technologies, Inc., Chicago, IL, USA), equipped with a linear transducer with a frequency range of 12-15 MHz. The probe was placed transversely to the anterior distal part of a supported forearm. The PLT was visualized anterior to the median nerve, medial to the flexor carpi radialis, and superficial to the flexor retinaculum, as seen in Figure 2. Confirmed absence was noted accordingly if no indication of the PLT could be made, as seen in Figure 3.

As participants made their way through each of the PE maneuvers onto the ultrasound station, they carried their participant questionnaires with them. This was done so that the researchers could fulfill their role of inputting presence or absence in the designated section of the sheet. It is worth noting that the questionnaire was not pilot-tested due to the small sample size and straightforward questions. This protocol was used for each member of the study and ensured efficiency while maintaining confidentiality between participants and researchers. Each station had one researcher responsible for carrying out the task. However, for the ultrasound station, a secondary researcher was present to clarify any ambiguities (if present) for the primary researcher who handled the probe and made the final call. Finally, the last member of the study had the role of overseeing operations to ensure participants were well navigated and that all stations were running smoothly.

At the end of the data collection, which lasted two sessions of around two to three hours, all of the data from the participant forms were compiled into an Excel sheet using Microsoft Excel (Microsoft® Corp., Redmond, WA, USA), before being run through IBM SPSS Statistics for Windows, Version 28 (Released 2021; IBM Corp., Armonk, NY, USA) to derive statistical significance. Chi-square and one-sample t-tests were performed to confirm the significance of the results, with sample sizes and degrees of freedom calculated. The prevalence of the muscle was compared between the physical exam and ultrasound through both the presence in total wrists and bilateral/unilateral identification.

## Results

This study aimed to investigate the detection of the PLT using various techniques, focusing on potential correlations with gender, ethnicity, and the dominant hand. It also sought to identify the physical examination test - whether Mishra I, Schaeffer, or Thompson - that displayed the highest accuracy relative to ultrasound in detecting the presence of the PLT. Ultrasound served as the reference standard, and its results were compared with the three physical examination methods through a Chi-square analysis in Tables 1-3. Degrees of freedom and significance values are listed below for each test, and the p-values show a statistically significant association in all three physical examinations when compared to ultrasound.

**Table 1:** Statistics of Schaeffer's Test Association With Ultrasound A total of 61 participants were analyzed based on the detection of the palmaris longus muscle through bilateral presence, unilateral presence, or total absence. Statistical values for Schaeffer's test - including Chi-square analysis, likelihood ratio, and linear-by-linear association - are presented. Degrees of freedom are listed as "df." The two-sided asymptotic significance confirms a statistically significant association in all tests.

Test Name	Value	df	Asymptotic Significance (Two-Sided)
Pearson Chi-Square	49.064	4	< 0.001
Likelihood Ratio	48.655	4	< 0.001
Linear-by-Linear Association	31.610	1	< 0.001
No. of Valid Cases	61	-	-

**Table 2:** Statistics of Thompson's Test Association With Ultrasound A total of 61 participants were analyzed based on the detection of the palmaris longus muscle through bilateral presence, unilateral presence, or total absence. Statistical values for Thompson's test - including Chi-square analysis, likelihood ratio, and linear-by-linear association - are presented. Degrees of freedom are listed as "df." The two-sided asymptotic significance confirms a statistically significant association in all tests.

Test Name	Value	df	Asymptotic Significance (Two-Sided)
Pearson Chi-Square	59.422	4	< 0.001
Likelihood Ratio	53.120	4	< 0.001
Linear-by-Linear Association	31.575	1	< 0.001
No. of Valid Cases	61	-	-

**Table 3:** Statistics of Mishra I's Test Association With Ultrasound A total of 61 participants were analyzed based on the detection of the palmaris longus muscle through bilateral presence, unilateral presence, or total absence. Statistical values for Mishra I's test - including Chi-square analysis, likelihood ratio, and linear-by-linear association - are presented. Degrees of freedom are listed as "df." The two-sided asymptotic significance confirms a statistically significant association in all tests.

Test Name	Value	df	Asymptotic Significance (Two-Sided)
Pearson Chi-Square	47.306	4	< 0.001
Likelihood Ratio	45.630	4	< 0.001
Linear-by-Linear Association	27.099	1	< 0.001
No. of Valid Cases	61	-	-

One-sample t-tests were performed, as shown in Table 4, for the Schaeffer test, the Thompson test, the Mishra I test, and the ultrasound, against a test value of 0. All tests demonstrated statistically significant differences from the test value (all p < 0.001), with mean differences ranging from 1.21311 (Mishra I test) to 1.55738 (ultrasound). Consistent with these findings, one-sample effect sizes in the Table 5 analyses yielded consistently large effect sizes. Cohen's d values ranged from 1.300 (Mishra I test) to 2.038 (ultrasound), with corresponding Hedges' correction values closely aligned, and the 95% confidence intervals for all effect sizes did not include 0, reinforcing the robustness and substantial magnitudes of these observed differences. Specifically, the ultrasound test demonstrated the largest observed effect. 

**Table 4:** One-Sample T-test Results for All Methods of Detecting Palmaris Longus Tendon Values of the t-test for each test are listed, alongside degrees of freedom and significance values.

Test Name	Values				
	t	df	Significance		Mean Difference
			One-Sided p-value	Two-Sided p-value	
Schaeffer Test	11.284	60	< 0.001	< 0.001	1.26230
Thompson Test	11.229	60	< 0.001	< 0.001	1.29508
Mishra I Test	10.155	60	< 0.001	< 0.001	1.21311
Ultrasound	15.915	60	< 0.001	< 0.001	1.55738

**Table 5:** Effect Size Results of Physical Examination Methods and Ultrasound Effect size analysis included Cohen's d and Hedges' correction values, with all estimates falling within the 95% confidence interval.

Test Name		Standardizer	Point Estimate	95% Confidence Interval	
				Lower	Upper
Schaeffer Test	Cohen's d	0.87372	1.445	1.082	1.802
	Hedges' Correction	0.88484	1.427	1.068	1.779
Thompson Test	Cohen's d	0.90082	1.438	1.076	1.794
	Hedges' Correction	0.91228	1.420	1.062	1.771
Mishra I Test	Cohen's d	0.93300	1.300	0.955	1.639
	Hedges' Correction	0.94487	1.284	0.943	1.618
Ultrasound	Cohen's d	0.76430	2.038	1.593	2.477
	Hedges' Correction	0.77402	2.012	1.573	2.445

**Figure 4:**
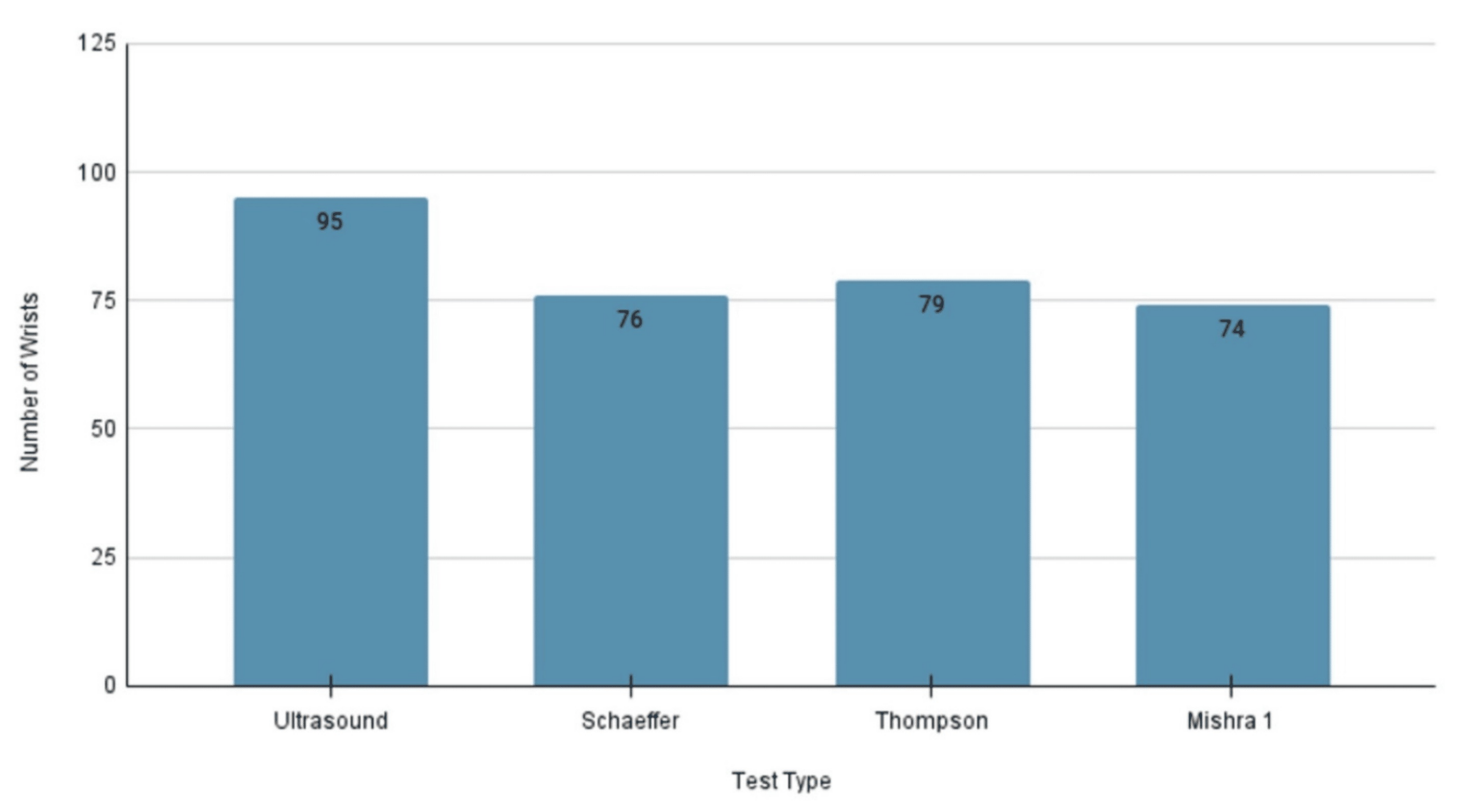
Presence of the Palmaris Longus Tendon Count of wrists where the palmaris longus tendon presence was detected by ultrasound, and the three physical examination methods.

A comprehensive examination of 122 wrists across 61 participants was conducted using four distinct methods to determine the presence of the PLT, as depicted in Figure 4. Utilizing the ultrasound method on all 122 wrists revealed the muscle's presence in 95 cases, representing 77.9%. Similarly, Schaeffer's test identified the muscle in 77 wrists (63.1%), Thompson's test in 79 wrists (64.8%), and Mishra I's test in 74 wrists (60.7%).

**Figure 5:**
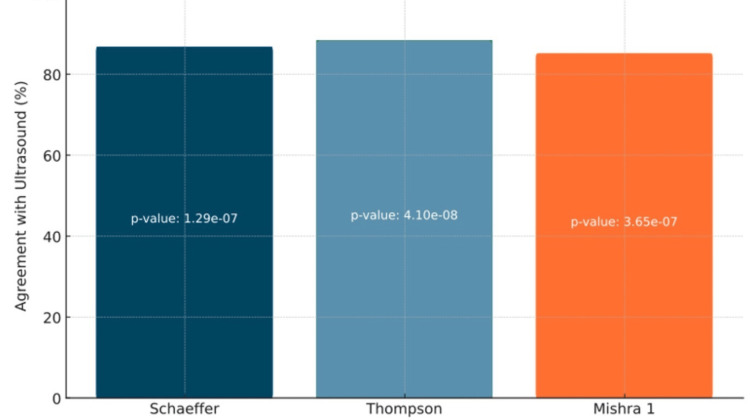
Comparison of Detection Methods With Ultrasonography on the Right Side This graph depicts the accuracy of each physical examination method when compared with ultrasound. p-values were added on the bars of each method to indicate significance. This data is specific to the right wrist.

**Figure 6:**
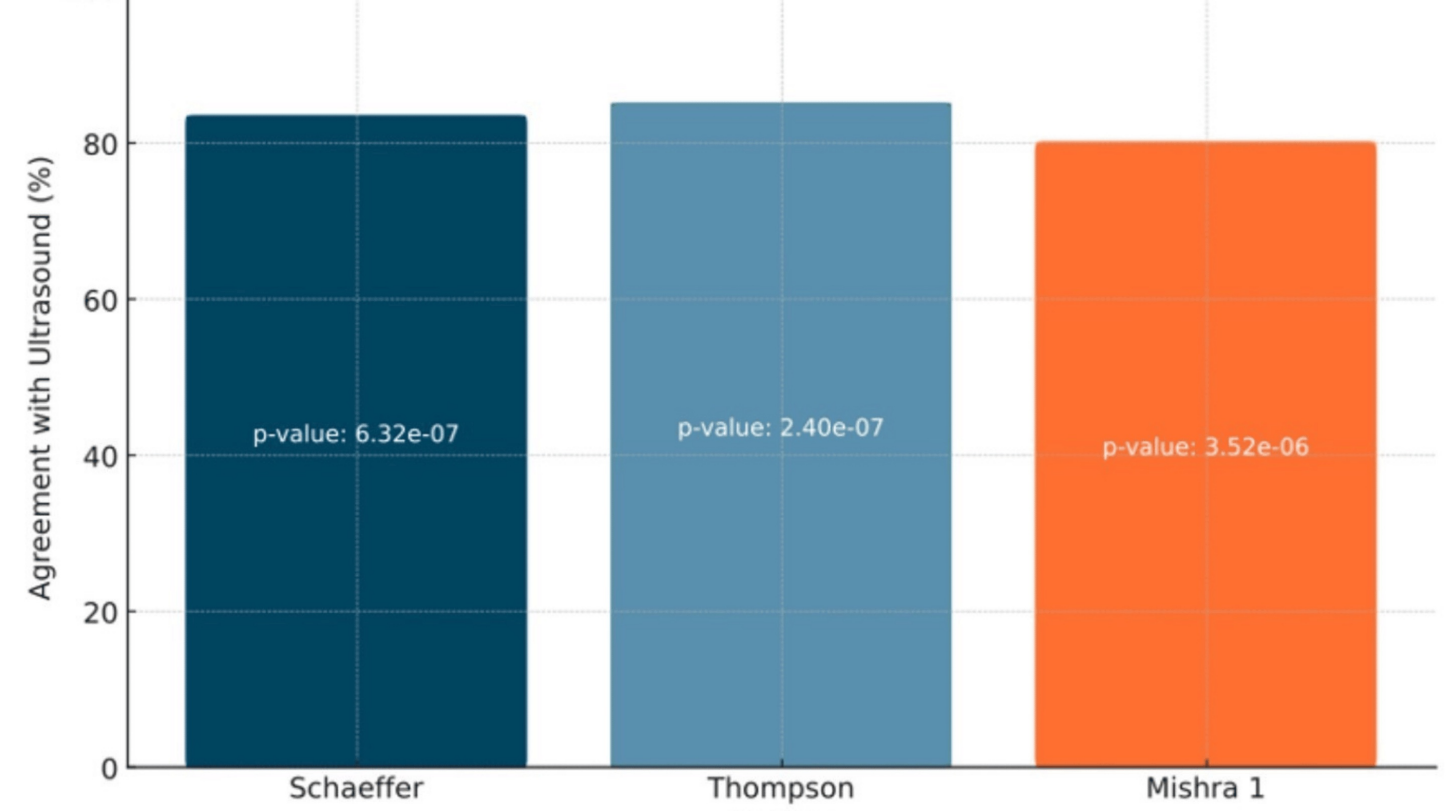
Comparison of Detection Methods With Ultrasonography on the Left Side This graph depicts the accuracy of each physical examination method when compared with ultrasound. p-values were added on the bars of each method to depict significance. This data is specific to the left wrist.

Figures 5-6 illustrate the comparison between ultrasound, the recognized gold standard method, and three physical examination tests: Schaeffer, Thompson, and Mishra I. The statistical analysis uncovered significant differences. On the right side, Schaeffer's, Thompson's, and Mishra I's tests exhibited substantial differences, with p-values of 1.29 × 10^-7^, 4.10 × 10^-8^, and 3.65 × 10^-7^, respectively. The p-values for the left side were 6.32 × 10^-7^ for Schaeffer, 2.40 × 10^-7^ for Thompson, and 3.52 × 10^-6^ for Mishra I.

This study had 61 participants, 33 of whom were female and 28 were male. An examination of the data by gender revealed that the right hand in females showed a higher incidence of positive detections, whereas in males, the left hand demonstrated more frequent positive results. Despite these observed differences, statistical analysis did not find any significant differences between the genders for either hand. Furthermore, while the presence of the PLT varied among different ethnic groups, the analysis did not uncover any significant relationships favoring or opposing the presence of the PLT. Lastly, statistical analysis did not yield any significant correlation between hand dominance and the prevalence of the muscle.

Table 6 evaluates several methods for examining the PLT's presence and absence. The Schaeffer test recorded bilateral presence in 54.10% (n = 66), bilateral absence in 27.87% (n = 34), and unilateral presence or absence in 9.02% (n = 11). The Thompson test resulted in 59.02% (n = 72) bilateral presence, 29.51% (n = 36) bilateral absence, and 5.74% (n = 7) unilateral presence or absence. The Mishra I test observed 55.74% (n = 68) bilateral presence, 34.43% (n = 42) bilateral absence, and 4.92% (n = 6) unilateral presence or absence. 

**Table 6:** Presence and Absence of the Palmaris Longus by Physical and Sonographic Methods This table shows the bilateral and unilateral presence and absence of the palmaris longus, with each physical examination method listed separately and their combined averages presented together for comparison with ultrasound. Unilateral presence and absence values are given in the last column; for example, in Schaeffer's test, 11 wrists were unilaterally present, and 11 were unilaterally absent.

Test	Bilateral Presence (n, %)	Bilateral Absence (n, %)	Unilateral Presence/Absence (n, %)
Schaeffer	66 (54.10%)	34 (27.87%)	11 (9.02%)
Thompson	72 (59.02%)	36 (29.51%)	7 (5.74%)
Mishra I	68 (55.74%)	42 (34.43%)	6 (4.92%)
Combined Physical Exams	68.66 (56.28%)	37.33 (30.60%)	8 (6.56%)
Ultrasound	88 (72.13%)	20 (16.39%)	7 (5.74%)

**Figure 7:**
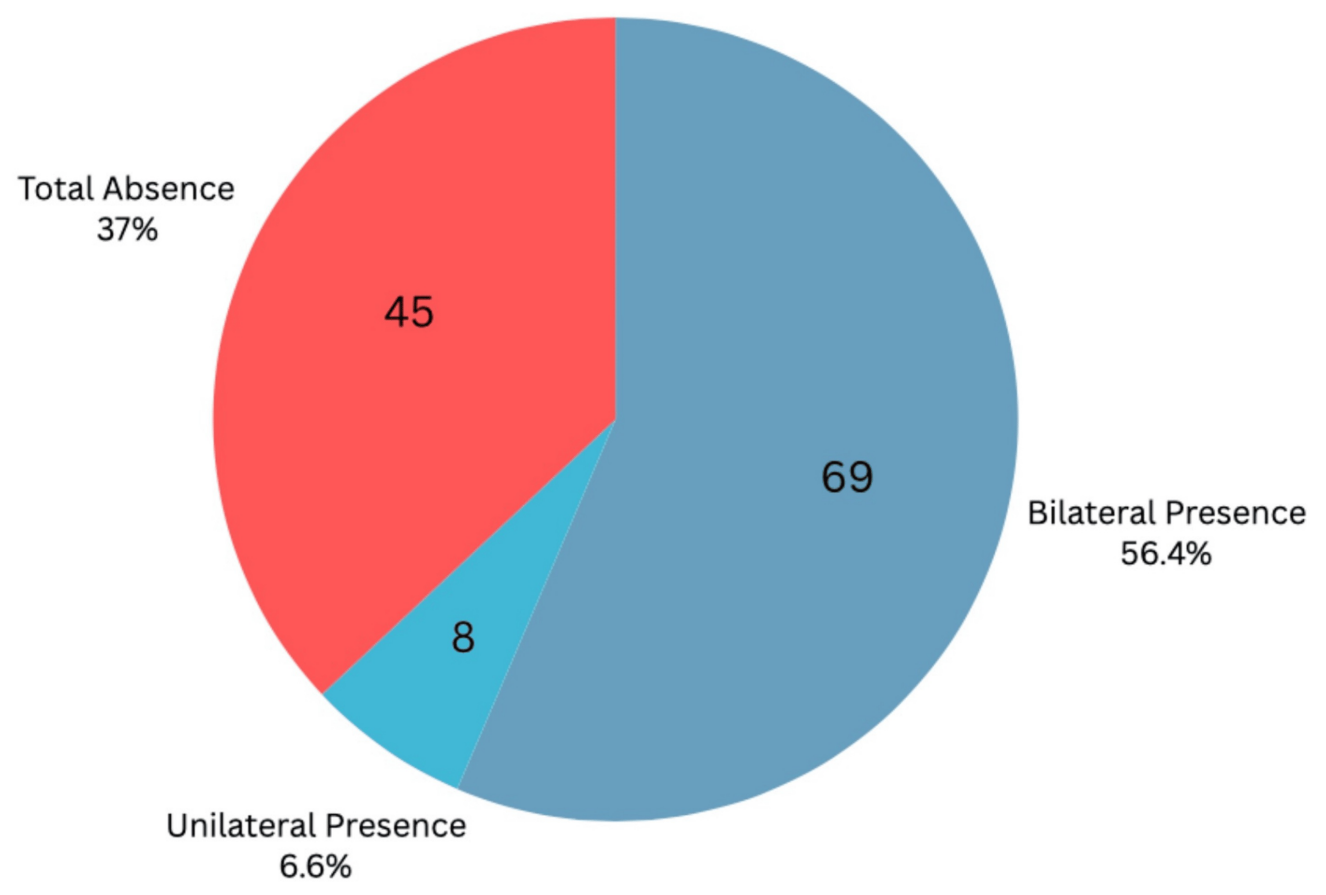
Combined Physical Exam Average Prevalence of the Palmaris Longus This pie chart shows the palmaris longus detection in participants by the combined physical examination methods. Average count values are rounded to the nearest whole number.

**Figure 8:**
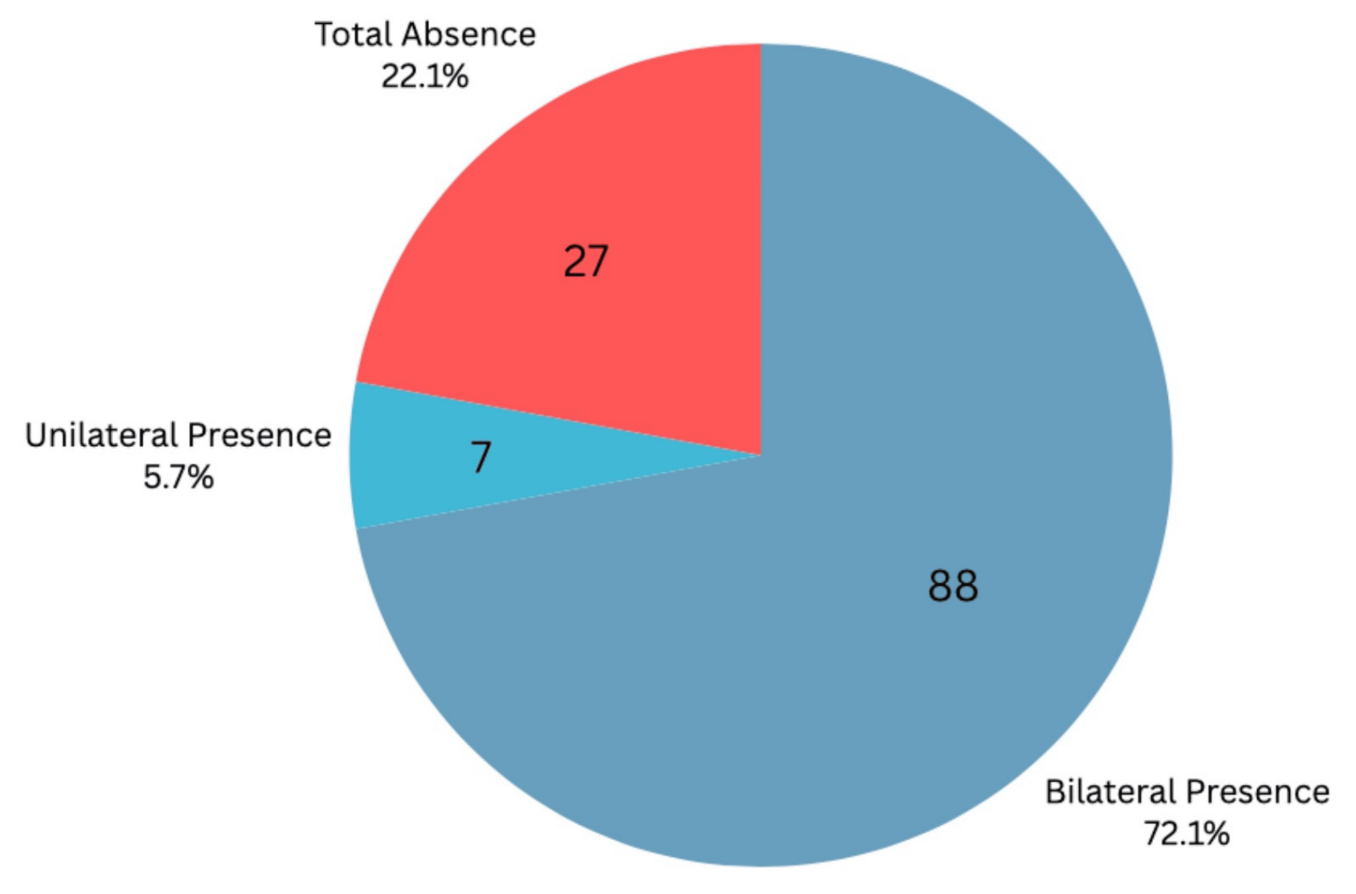
Ultrasonography Prevalence of the Palmaris Longus This pie chart shows palmaris longus detection in participants by ultrasound.

As illustrated in Figures 7-8, taking into account that unilateral absence is grouped with total absence, we conclude that on ultrasound examination, the PLT was detected bilaterally in 72.13% of wrists (n = 88), unilaterally in 5.74% (n = 7), and was absent in 22.13% (n = 27) of the 122 wrists examined. Physical examination methods detected an average of 56.28% bilaterally (n = 68.66), 6.56% unilaterally (n = 8), and 37.16% absent (n = 45.33). Total detection by ultrasound was 77.87% (n = 95), while the physical examination average was 62.84% (n = 76.66).

## Discussion

While functionally dispensable, the PLT holds significant importance due to its frequent use in tendon grafting procedures as an accessible donor site, owing to its superficial location and a tendon length of around four to six inches, similar to the plantaris tendon [6]. However, this tendon demonstrates a series of anatomical anomalies, including agenesis, anomalous origin, bifid, and reversed morphologies [7]. This highlights the need for detection methods to rule out agenesis and confirm the presence of the muscle, which is obtained through clinical and radiological examinations. Although the majority of previous studies of the PLT use physical examination methods solely for results, it is essential to establish the accuracy of that approach in contrast with sonographic measures, the latter of which has shown significantly more accurate findings [8]. This study compares the accuracy of physical examination methods to that of ultrasonography. 

The PLT was identified using four methods in total: ultrasonography and the three physical examination methods - Schaeffer, Thompson, and Mishra I. Ultrasound demonstrated the presence of the tendon in 77.9% of wrists, or collectively in 95 out of the 122 wrists examined. In contrast, the three physical examination methods recognized the presence with much less accuracy. The first station, Schaeffer’s test, revealed the presence in 63.11% of wrists (77 out of 122). The second station, Thompson’s test, detected the muscle in 64.75% of wrists (79 out of 122). Lastly, the Mishra I test revealed the presence of the muscle in 60.66% of wrists (74 out of 122). Moreover, although not all presence was bilateral, sonographic examination confirms that 72.13% of participants had bilateral presence (n = 88), and 5.74% were unilateral (n = 7), which equates to 83.6% of participants having at least one PLT, with the rest possessing bilateral PLT agenesis. Tendon presence being the majority is concurrent with our expected findings, where we hoped to observe popularity in the prevalence of the muscle. 

In evaluating the presence of the PLT across various demographic factors, our findings revealed slight differences in laterality between genders; females more frequently exhibited tendon presence in the right hand, while males showed a higher incidence in the left. However, these trends were not statistically significant, indicating that gender is not a reliable predictor of tendon presence. Similarly, although variations were observed across different ethnic groups, no statistically significant associations were found, preventing any conclusions regarding ethnicity as a determining factor. Hand dominance was also analyzed, but the results demonstrated no significant correlation between dominance and tendon prevalence. These findings support previous literature that similarly reported no association between the presence of the PLT and gender [1]. This falls opposite to other publications, where gender was implicated as a factor in PLT prevalence, such as females having a higher percentage of absence of the tendon [2]. Altogether, our results suggest the degree of individual anatomical variation and reinforce the importance of direct clinical assessment over demographic assumptions when evaluating the PLT.

Ultrasonography served as the confirmatory tool in this study, following the application of physical examination techniques. While the three physical tests (Thompson, Schaeffer, and Mishra I) demonstrated varying degrees of effectiveness, they consistently underperformed in terms of accuracy when compared to ultrasound. Among them, Thompson's test yielded results closer to the ultrasound findings, followed by Schaeffer’s test, with Mishra I showing the least accuracy. These differences highlight the limitations of relying solely on physical maneuvers, which are subject to potential variability in technique and anatomical interpretation. In contrast, ultrasound provided a direct and reliable visualization of the PLT, particularly in its anatomical location superficial to the flexor retinaculum, which further fortifies the idea of its simplicity to locate. As such, ultrasonography emerges as a superior, non-invasive diagnostic tool that not only minimizes the risk of false negatives but is also widely accessible in clinical settings. Although cadaveric dissection remains the definitive method for confirming tendon presence, it is not practical in everyday medical practice, making ultrasound the most efficient and accurate alternative.

As previously mentioned, the PLT is relevant to surgical fields in tendon grafting. By combining physical examination methods and ultrasonography, physicians can confirm its presence, location, and characteristics to enhance the utilization of its tendon. In contrast, physical examination methods alone are not likely to provide information on morphologies outside the scope of agenesis, unlike ultrasonography. In this case, the use of ultrasound may be implemented further as a combined approach and, therefore, ensure the accuracy of its existence and additionally provide further insight into its anatomy. 

Comparison with previous studies

This investigation distinguishes itself from comparable studies that aim to identify the presence of the PLM through diverse methodologies. Unlike existing literature, this study adopts a distinctive approach by employing a combined method for detecting the muscle. Evidently, reliance solely on physical examination methods has been demonstrated to be less reliable. Therefore, while preceding studies have primarily utilized physical examination techniques, our research also incorporates confirmatory ultrasound following the initial physical examination [8].

Similarly, a systematic review in 2013 exclusively relied on studies utilizing physical examination methods for recognizing the muscle in the forearm, without using ultrasonography [9]. This raises questions about the reliability of demographic data collected from large samples that likely underwent only physical examination. In more detail, since physical examination methods possess a higher false negative rate, the presence of this muscle is likely to be underestimated, and specific demographic claims may be affected.

Ultrasound was also mentioned in other articles using cadaveric data, where 100% sensitivity and specificity were reported, reinforcing its accuracy in detection [10]. Other imaging modalities included an MRI-based study in PLT identification; however, MRI is likely to be more expensive and time-consuming to perform, unlike ultrasound [11].

Interestingly, the PLM was confirmed as a hereditary trait, as indicated by research using Schaeffer’s test among families [12], possibly due to the accessibility of these maneuvers. Regardless, ultrasound should become a more accessible resource in the clinical setting to increase the accuracy of results, as it is seen as a reliable tool in assessing skeletal muscles such as the PLM [13].

Regarding its use, the PLM has been deemed an adequate grafting site with sufficient width, with further predictive values of its properties based on forearm length [14]. In terms of muscle presence, multiple cadaveric studies demonstrate similar prevalence statistics - for example, 25% of PLMs were reported as missing from upper extremities [15]. Further data collection trials should be considered for a more extensive and accurate analysis, aided by demographic factors, to support more precise associations and deepen understanding of its variability.

Limitations and future directions

Despite its strengths, this study has several limitations. Firstly, the sample, although adequate for primary analysis, was not sufficiently large to detect differences across ethnic subgroups, due to both diversity density and size. Secondly, examiners were assigned to a single station for the entirety of the course, which provided consistency in station sampling; however, this introduces the potential for measurement bias. Third, the study did not assess tendon morphology or dimensions, which could provide data indicating that, in certain patients, the tendon - though present - may be unsuitable for grafting, thus reducing its clinical significance. 

Future studies should utilize ultrasound to explore inter-observer reliability in ultrasonographic detection, investigate and correlate the tendon’s morphometric characteristics, and provide a larger, denser sample size to identify more accurate demographic analytics. Additionally, including cadaveric validation would further strengthen the accuracy of anatomical findings, increasing the validity of sonographic measures.

## Conclusions

This study demonstrated that ultrasonography is a significantly more accurate and reliable method for detecting the presence of the PLT compared to physical examination techniques. Among the physical tests, Thompson's method showed the highest accuracy. However, all three techniques (Schaeffer, Thompson, and Mishra I) consistently underperformed relative to ultrasound. While the overall prevalence of the tendon was high, and bilateral presence was the most common, no statistically significant associations were found with gender, ethnicity, or hand dominance. These results emphasize the variability of human anatomy and reinforce the limitations of relying solely on physical examination. Given its simplicity, accessibility, and diagnostic precision, ultrasound should be integrated into clinical assessments of the PLM - especially in contexts where accurate identification is essential, such as preoperative planning for tendon grafting. Future research should explore tendon morphology and sample larger, more diverse populations through sonography to better understand demographic patterns and validate ultrasound as a standard diagnostic approach.

## Appendices

Participant questionnaire

PROJECT TITLE: A Comparative Study of the Presence of the Palmaris Longus Tendon Using Physical and Ultrasound Examination

Participant number: _________________________________________

The information you provide will be treated confidentially. There is no known potential harm for the measurements we will obtain from you. Please fill in the following form:

Age: ________

Gender:

o Male 

o Female

o Other

Dominant Hand: 

o Right-Handed

o Left-Handed

o Ambidextrous

Have you ever had a surgical procedure involving your hands or wrists? 

o No

o Yes, what was it?_____________________________________

Please select the best that describes your ethnic origin:

Choose ONE section from A to G, then tick the appropriate box to indicate your ethnic group:

A: White

o British

o Irish

o Any other White background (please specify) ____________________

B: Mixed

o White and Black Caribbean

o White and Black African

o White and Asian

o Any other mixed background (please specify) ______________________

C: Asian or Asian British

o Indian

o Pakistani

o Bangladeshi

o Chinese

o Any other Asian background (please specify) ______________________

D: Black or Black British

o Caribbean

o African

o Any other Black background (please specify) ______________________

E: Arab

F: Others: please specify: _____________________________________________________________________________________________________________________________

------------------------------------------------------------------------------------------------------------------------------------

FOR RESEARCHER USE ONLY

1) Right Wrist

Schaeffer’s Test

o Positive

o Negative

Thompson’s Test

o Positive

o Negative

Mishra I’s Test

o Positive

o Negative

Ultrasound Imaging

o Positive

o Negative

2) Left Wrist

Schaeffer’s Test

o Positive

o Negative

Thompson’s Test

o Positive

o Negative

Mishra I’s Test

o Positive

o Negative

Ultrasound Imaging

o Positive

o Negative
